# Supplementation of Dietary Crude Lentinan Improves the Intestinal Microbiota and Immune Barrier in Rainbow Trout (*Oncorhynchus mykiss*) Infected by Infectious Hematopoietic Necrosis Virus

**DOI:** 10.3389/fimmu.2022.920065

**Published:** 2022-06-22

**Authors:** Guangming Ren, Liming Xu, Jingzhuang Zhao, Yizhi Shao, Xiaoyu Chen, Tongyan Lu, Qiya Zhang

**Affiliations:** ^1^ Department of Aquatic Animal Diseases and Control, Heilongjiang River Fisheries Research Institute, Chinese Academy of Fishery Sciences, Key Laboratory of Aquatic Animal Diseases and Immune Technology of Heilongjiang Province, Harbin, China; ^2^ State Key Laboratory of Freshwater Ecology and Biotechnology, Institute of Hydrobiology, Chinese Academy of Sciences, Wuhan, China; ^3^ Technology Center of Wuhan Customs, Wuhan, China

**Keywords:** crude lentinan, IHNV, intestinal microbiota, SCFAs, immune barrier, tight junction–related genes

## Abstract

The effects of crude lentinan (CLNT) on the intestinal microbiota and the immune barrier were evaluated in rainbow trout (*Oncorhynchus mykiss*) infected by infectious hematopoietic necrosis virus (IHNV). The results showed that supplementary CLNT declined the rainbow trout mortality caused by IHNV, which suggested that CLNT has preventive effects on IHNV infection. IHNV destroyed intestinal integrity, as well as caused the intestinal oxidative and damage in rainbow trout. Supplementary CLNT significantly strengthened the intestinal immune barrier by declining intestinal permeability, as well as enhancing intestinal antioxidant and anti-inflammatory abilities in IHNV-infected rainbow trout (*P*<0.05). In addition, CLNT modified the aberrant changes of intestinal microbiota induced by IHNV, mainly represented by promoting the growths of *Carnobacterium* and *Deefgea* and inhibiting *Mycobacterium* and *Nannocystis*. Especially, supplementing with CLNT significantly promoted the growth of short-chain fatty acid–producing bacteria (*P*<0.05) and consequently increased the production of acetic acid, butanoic acid, and hexanoic acid in the intestine of IHNV-infected rainbow trout. Furthermore, it was speculated that CLNT could regulate the self-serving metabolic pathways of intestinal microbiota induced by IHNV, such as fatty acid metabolism and amino acid metabolism. Together, CLNT played the antiviral effects on IHNV infection through strengthening the intestinal immune barrier, as well as regulating intestinal microbiota and SCFA metabolism in rainbow trout. The present data revealed that CLNT exerted a promising prebiotic role in preventing the rainbow trout from IHNV infection.

## Introduction

Currently, aquaculture has expanded globally with the increases in the production and the number of fish species being cultured. The intensive rearing conditions in fish farms provide new opportunities for the rapid spread of aquatic pathogenic microbes. Infectious hematopoietic necrosis virus (IHNV) is the causal agent of a highly contagious disease that influences many species of salmonid fishes, particularly rainbow trout (*Oncorhynchus mykiss*) and sockeye salmon (*O. nerka*) (1). It belongs to the genus *Novirhabdovirus* within the family Rhabdoviridae and is a negative-strand RNA virus with a genome of approximately 11 kb that encodes six proteins: nucleocapsid (N), polymerase-associated phosphoprotein (P or M1), a matrix protein (M or M2), surface glycoprotein (G), non-virion protein (NV), and virus RNA polymerase (L) (2). IHNV can induce the mortality of 80%~100% to the fry and juveniles of salmonidae (3, 4). Meanwhile, those fish that survive an IHNV infection might become the asymptomatic carriers of the virus for a long period and lead to vertical transmission to the susceptible species, which causes a more widespread transmission and more severe hazard (3, 5). In recent years, the outbreaks of IHN frequently occurred in many provinces of China, which not only cause a huge economic loss but also block the industry development of rainbow trout. However, there are no vaccines or drugs commercially available against IHNV in China.

As an alternative to vaccines, the application of immunostimulants is a worthy strategy to enhance the immune status of fish and reduce the severity of infectious disease outbreaks (6, 7). Lentinan, as an immunostimulant, has diverse traits including anti-inflammatory, antioxidant, and antimicrobial abilities (8, 9). Several studies on lentinan have shown that it is helpful for protecting the host from the pathogen attacks through enhancing the host immunity (10–12). Furthermore, several researchers have proved that dietary lentinan played important roles in reshaping intestinal microbiota to relieve intestinal damages (8, 11–13). As we commonly know, IHN viruses can replicate in large numbers in the intestine and lead to the necrosis of intestinal wall cells in IHNV-infected rainbow trout (5, 14). The main pathognomonic lesion of intestine caused by IHNV is manifested by the yellow mucus in the intestine (15–17). This pathological damage could activate the immune responses of intestine in IHNV-infected rainbow trout (18–20). Similar to mammalian, intestinal mucosal immunity is the first line in fish to defense viral invasion, which played important roles in regulating both intestinal microbiota homeostasis and pathogen control at mucosal sites (21, 22). Moreover, virus-induced alterations of intestinal microbiota could influence the metabolic phenotype of the host, and the production of microbes associated metabolites and derivatives of intestinal microbes, such as SCFAs (23, 24). These metabolites act as messengers of the intestinal microbes to regulate together the host immunity (25, 26). Obviously, it is beneficial to remodel the aberrant intestinal microbiota for ameliorating the host inflammatory injury.

We previously found that lentinan had inhibitory roles in IHNV replication *in vitro (* 27). However, its antiviral role and the corresponding mechanism *in vivo* are unclear. This study aims to explore the preventive roles of crude lentinan (CLNT) on IHNV infection *in vivo*. Moreover, its effects on the intestinal microbiota, intestinal immune barrier, and SCFA metabolism were investigated in IHNV-infected rainbow trout. The results will provide practical data for exploiting the efficient immunostimulant to protect the rainbow trout from IHN detriment.

## Material and Methods

### Materials

CLNT was obtained from the body of *Lentinula edodes* by hot water extraction and ethanol precipitation with a yield of 7.51%. In brief, the powder of the dried body was suspended in the distilled water at 60°C for 2.50 h. The supernatant was concentrated in a rotary evaporator under reduced pressure, purified with the Sevage reagent, then dialyzed in a 500~1,000 D dialysis bag and lyophilized for obtaining the CLNT. CLNT powder is soluble in water and insoluble in ethanol, acetone, and ether. The total soluble sugar percentage of CLNT is 51.88%.

Assay kits for superoxide dismutase (SOD), total antioxidant capacity (T-AOC), malondialdehyde (MDA), and total protein were purchased from Nanjing Jiancheng Bioengineering Institute (Nanjing, China). The Fecal DNA Isolation Kit and DNA Purification Kit were purchased from Tiangen Biotechnology Co. Ltd. (Beijing, China). The TRIzol reagent was purchased from Invitrogen Co. Ltd (Carlsbad, CA, USA). The One-Step TB Green PrimeScript RT-PCR PLUS Kit was purchased from Takara Bio Inc. IHNV-sn1203 was isolated from diseased rainbow trout and stored in our laboratory as previously described (28).

### Diet Preparation

The compositions of the basal diet are shown in **Table 1**. The formulation has been shown to be nutritionally adequate for the growth requirement of juvenile trout. CLNT was supplemented into the basal diet to formulate the diets of the low dosage of CLNT (1.0%) and the high dosage of CLNT (2.0%). Briefly, all the ingredients were ground into powder and added with 1.0% and 2.0% of CLNT. Water was gradually added to the mixed ingredients with a proportion of 40% (water/mixed ingredients). Subsequently, the obtained mixture was made into 1.5 mm pellets using a meat grinder and then air-dried to below 100 g/kg moisture of diet. After drying, the obtained sinking diets were sealed in bags and stored at -20°C until used.

**Table 1:** Compositions of the basal diet (g/kg) for rainbow trout.

Ingredients	Basal diet
Fish meal	300
wheat meal	200
Corn gluten meal	135
Soy protein concentrate	200
Fish oil	70
Soybean oil	50
Cellulose	8.0
Mineral premix^a^	20
Vitamin mixture^b^	10
Dimethyl-β-propiothetin	2.0
Choline chloride	5.0
Chemical composition	%
Crude protein	48.30
Crude lipid	13.80

a Mineral premix (mg/kg mixture) MgSO_4_·7H_2_O, 100.00, NaH_2_PO_4_·2H_2_O, 300.00, KCl, 125.00, ferric citrate, 50.00, ZnSO_4_·H_2_O, 75.00, Ca-lactate, 100.00; CuSO_4_, 15.00; FeCl_3_, 25.00, Na_2_SeO_3_, 10.00; MnSO_4_·H_2_O, 15.00, and CoCl_2_·6H_2_O, 10.0.

b Vintamin premix (mg/kg mixture) L-ascorbic acid, 300.00, tocopheryl acetate, 300.00, thiamin, 15.00, riboflavin, 30.00, pyridoxine, 15.00 niacin, 175.00, calcium pantothenate, 200.00, inositol, 300.00, folic acid, 50.00, menadione, 200.00, and cyanocobalamin, 50.00.

### Animals and Treatments

Animal experiments were performed in compliance with the relevant laws approved by the Animal Welfare Committee of Heilongjiang River Fishery Research Institute, Chinese Academy of Fishery Sciences. All efforts were made to minimize animal suffering. Rainbow trout (body weight 10.00 ± 2.00 g) were acclimatized for 14 days. Water at a constant temperature of 15 ± 0.2°C was supplied in a flow-through system (water inflow rate: 8 L/min), and the dissolved oxygen level ranged from 7.50 to 8.50 mg/L. Fish were randomly assigned into four groups of 60 each, including the normal control group (NC), challenge group (IH), low-dosage group (LD), and high-dosage group (HD). Each group was randomly assigned to triplicate. Fish both in NC and IH groups were fed with the basal diet, and the LD and HD groups were fed with the low dosage and high dosage of CLNT, respectively. All fish in four treatments were hand-fed twice daily (09:00 and 16:00) at 2.00% of body weight. Fish were fed for 28 days and then fasted for 1 day. Afterwards, fish in IH, LD, and HD groups were intraperitoneally injected with IHNV (100 TCID_50,_ 50 μl) and continuously observed for 14 days. In the NC group, fish were intraperitoneally injected with the same volume of sterile physiological saline. During the trial, the number of dead fish was recorded every day. At the end of the experiment, fish were anesthetized with 100 mg/L eugenol (Shanghai Reagent, Shanghai, China). Fecal samples from the intestinal tract were collected using sterile tweezers for DNA extraction and SCFA analysis. The partial small intestines were excised and fixed for histological and biochemical analysis, and the remains were homogenized for extracting RNA.

### Histological and Biochemical Analysis

The intestines were harvested and fixed in neutral buffered formalin for 24 h at room temperature. Subsequently, the samples were gradually dehydrated in graded ethanol concentrations and embedded in paraffin wax, sectioned, and stained with hematoxylin–eosin (H&E). The tissue sections were observed and photographed using a microscope (Leica DMi8, Leica, Germany).

The intestinal tissues were homogenized with ice-cold saline and centrifuged, and the supernatant was used to determine the levels of T-AOC, SOD, MDA, and total protein according to the manufacturer’s instructions. Each sample was analyzed in triplicate.

### Real-Time Quantitative PCR

Total RNA was extracted from intestines using the TRIzol reagent. RT-qPCR experiment was performed to detect the expressions of immune-related genes, as well as IHNV-N and IHNV-L genes using the Applied Biosystems 7500 System (Bio-Rad, Hercules, CA, USA) in triplicate. The primers of these genes were designed and showed in **STable 1**. β-Actin was used as an internal control to normalize the gene expression levels. The fold changes of mRNA expression were normalized to the level of the NC group by the comparative cycle threshold (Ct) method (2^−ΔΔCt^).

### Quantification of Short-Chain Fatty Acids

The obtained feces were added with 0.5% phosphate, thoroughly homogenized, and centrifuged to collect the supernatant. After adding the same amount of ethyl acetate, the mixtures were homogenized and centrifuged to collect the supernatant. Subsequently, the obtained supernatant was mixed with tetramethyl pentanoic acid, homogenized, and kept on ice for quantifying SCFA contents using the gas chromatography–mass spectrometer (GC-MS; Agilent 7890A/5975C).

### Intestinal Microbiota Analysis

The V3~V4 hypervariable region of the 16S ribosomal RNA (rRNA) gene was amplified, and PCR products were purified by the DNA purification kit. After the preliminary quality filtering, the entire qualified DNA was used to construct a library on an Illumina Hiseq 2500 platform. Data were analyzed with QIIME and R software. Alpha diversity was calculated by the Ribosomal Database Project (RDP) pipeline at 97% sequence identity. Beta diversity on the obtained weighted UniFrac distance and Bray–Curtis distance were analyzed using the principal coordinates analysis (PCoA). The 16S rRNA gene sequence of the sample was mapped to the Kyoto Encyclopedia of Genes and Genomes (KEGG) database for annotation, and the abundance of metabolic pathways was predicted based on the phylogenetic investigation of communities by the reconstruction of unobserved states 2 (PICRUSt2).

### Statistical Analysis

All data were presented as the mean ± standard error of the mean (SEM) using SPSS 21.0 software (SPSS Inc., Chicago, IL, USA), and the graphs were made with Prism 5.0 software. Differences between groups were tested by the analysis of variance (ANOVA), followed by the two-tailed unpaired Student’s t-test. The difference was considered statistically significant when *P*<0.05. Correlations among the key gena and immune-related genes were determined on t-value analysis obtained from the redundancy analysis (RDA) and Spearman correlation analysis.

## Results

### Fish Data

**Figure 1:**
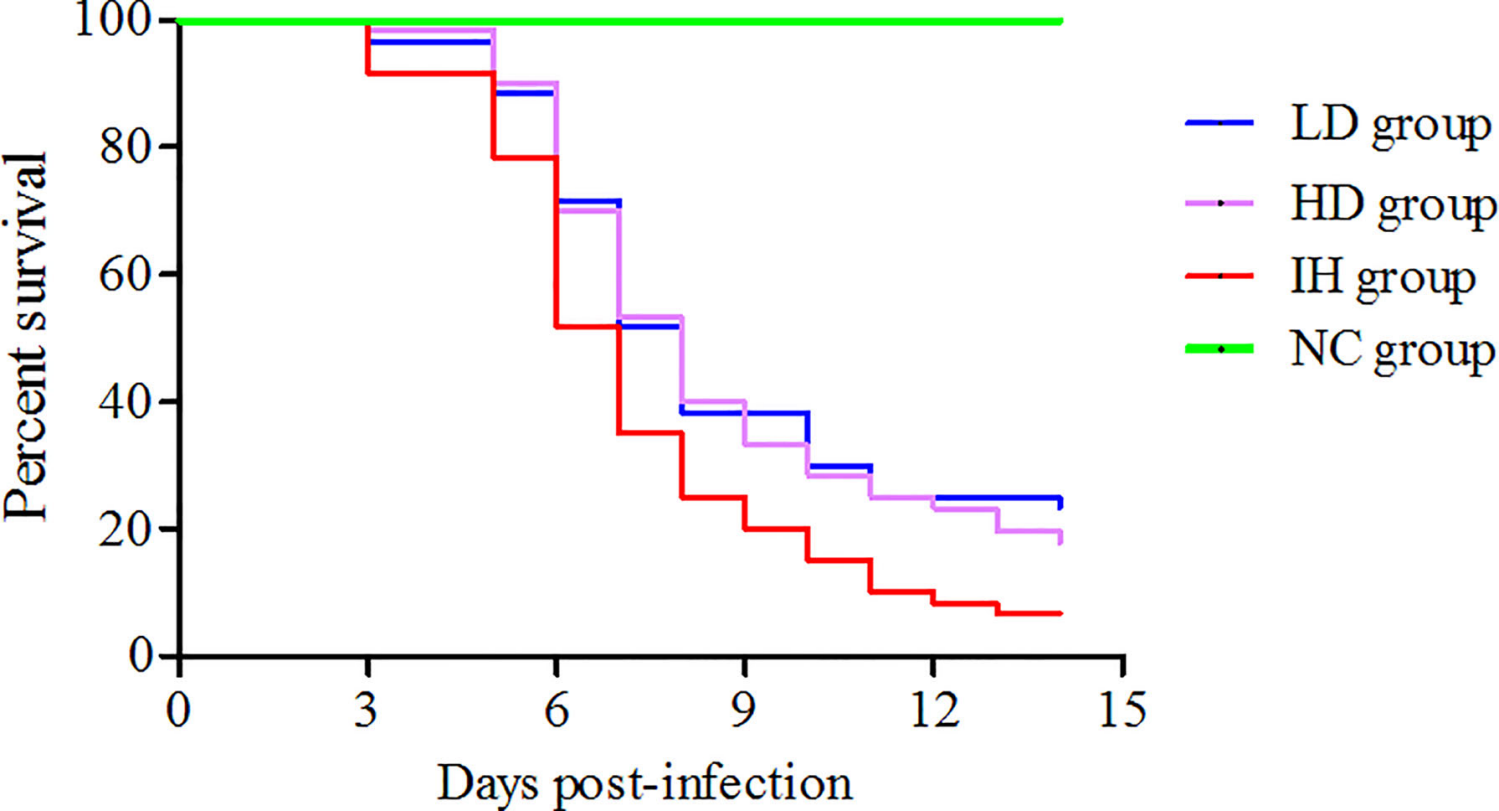
Survival curve of rainbow trout under the different treatments.

No mortality was recorded in the NC group during the experimental period (**Figure 1**). During the experiment, there was no significant difference in body weight among the groups (data were not shown). IHNV infection (IH group) induced the high mortality of rainbow trout (93.33%). Survival rates in LD and HD groups reached 23.33% and 17.71%, respectively. The results showed that CLNT had the preventive effects on IHNV infection in rainbow trout. Moreover, the protective effects of 1.0% dosage of CLNT were better than 2.0% dosage.

### Histological Observation

**Figure 2:**
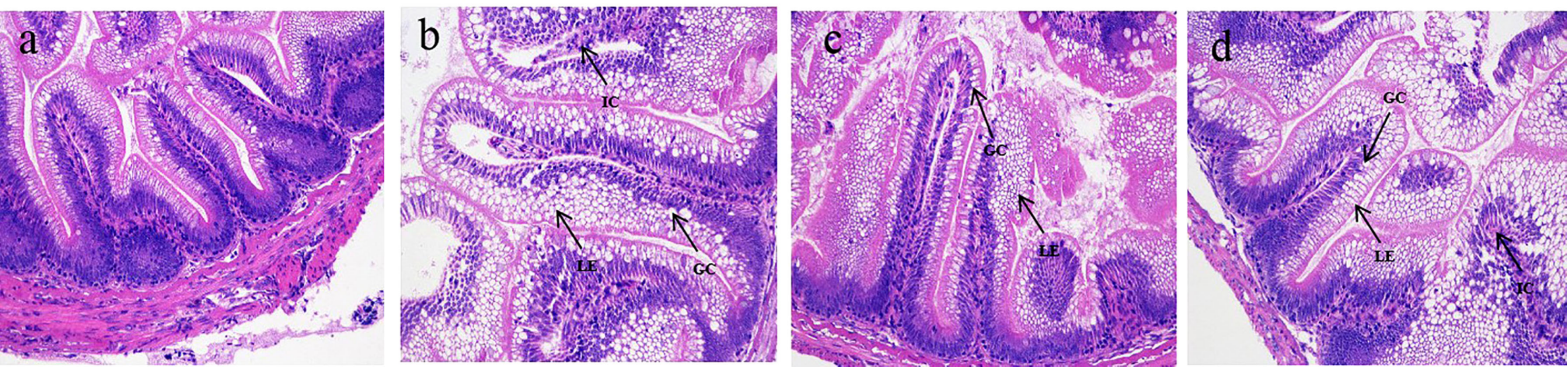
Histological changes of the intestine in rainbow trout. **(A–D)** The changes of intestinal architecture in normal control (NC), challenge (IH), low-dosage (LD), and high-dosage (HD) groups, respectively. ×200 magnifications; GC, LE, and IC were indicative of the goblet cell, lamina epithelialis, and inflammatory cell, respectively.

The normal intestinal architecture and cell structure were presented in the NC group, characterized by the intact intestinal mucosa and columnar epithelium arranged orderly in the well-organized intestine (**Figure 2A**). There were severe goblet cell hypertrophy, intra-epithelial leukocytosis, epithelium vacuolation, and inflammatory cell infiltration in the IH group (**Figure 2B**). Reversely, these symptoms were attenuated in rainbow trout supplemented by CLNT to some extent (**Figures 2C, D**). Supplementing with CLNT alleviated the pathological symptoms of intestine, especially low dosage of CLNT, which successfully attenuated the goblet cell hypertrophy, epithelium vacuolation, and lymphocyte infiltration.

### Effects of Crude Lentinan on Intestinal Antioxidant Activity

Intestinal T-AOC and SOD levels in the IH group were significantly declined compared with those in the NC group, and the MDA level significantly increased (*P*<0.05) (**Table 2**). The results showed that IHNV caused intestinal oxidative damage in rainbow trout. Intestinal T-AOC and SOD levels in LD and HD groups were 2.00 and 1.31 and 1.89 and 1.15 folds higher than those in the IH group, while MDA contents were 1.34 and 1.31 folds lower, respectively. The results indicated that supplementing with CLNT significantly elevated the intestinal antioxidative ability and then ameliorated the oxidative damage to intestine caused by IHNV in rainbow trout (*P*<0.05).

**Table 2:** Effects of supplementary 1% and 2% of crude lentinan (CLNT) on intestinal antioxidant ability in infectious hematopoietic necrosis virus (IHNV)–infected rainbow trout.

Items	NC group	IH group	LD group	HD group
T-AOC (mmol/g)	0.16 ± 0.03a	0.09 ± 0.003b	0.18 ± 0.04a	0.17 ± 0.05a
SOD (U/mgprot)	21.81 ± 2.10a	18.07 ± 3.64b	23.60 ± 2.44a	20.74 ± 1.13a
MDA (nmol/mgprot)	3.37 ± 0.72a	4.74 ± 0.49b	3.54 ± 0.55ac	3.62 ± 0.99c

Different letters indicated significant differences between groups (P < 0.05).

### Effects of Crude Lentinan on the Intestinal Immune Barrier

**Figure 3:**
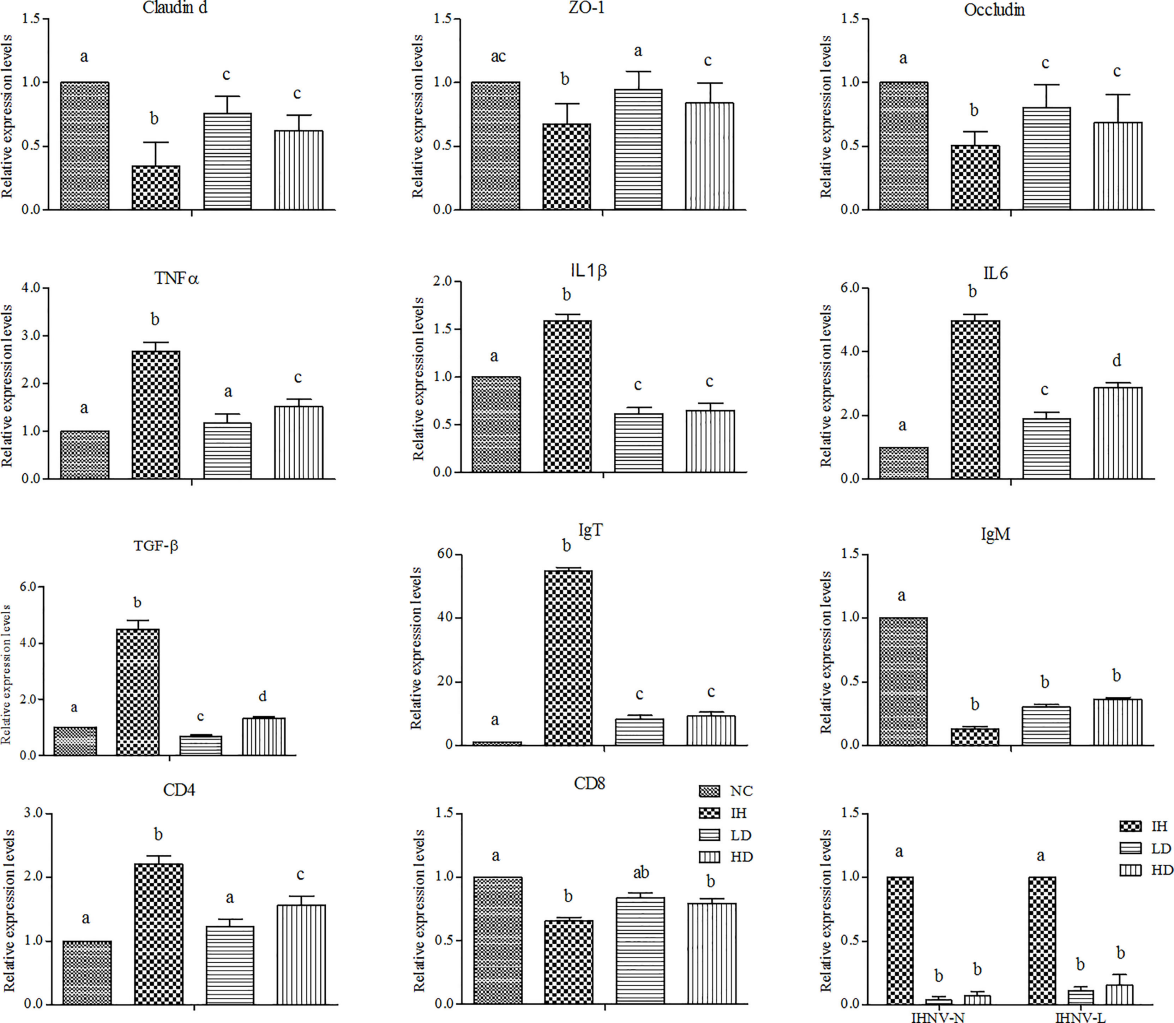
Changes in the expression of intestinal immune-related genes after dietary 1% and 2% of crude lentinan (CLNT) in infectious hematopoietic necrosis virus (IHNV)–infected rainbow trout (*P*<0.05). Different letters indicated significant differences among the groups.

IHNV infection significantly decreased the expression levels of claudin d, ZO-1, and occludin genes in the intestine compared with those in the NC group (*P*<0.05) (**Figure 3**). Supplementing with CLNT significantly increased their expression levels, of which in the LD group were 1.39, 1.60, and 2.18 folds higher than those in the IH group and in the HD group were 1.24, 1.37, and 1.78 folds higher (*P*<0.05). Furthermore, IHNV infection significantly increased the expression of intestinal tumor necrosis factor-α (TNF-α ), interleukin 1β (IL1β), interleukin 6 (IL-6), and transforming growth factor-β (TGF-β) genes (*P*<0.05). Supplementing with CLNT significantly decreased the expressions of these pro-inflammatory cytokines compared with those in the IH group (*P*<0.05). Furthermore, supplementing with CLNT increased the expressions of intestinal IgM and CD8 genes, presented by the higher levels than those in the IH group. Unexpectedly, the expression levels of IgT and CD4 genes in the IH group were significantly higher than those in the NC, LD, and HD groups, respectively (*P*<0.05). However, the expression levels of intestinal IHNV-N and IHNV-L in the LD and HD groups were significantly decreased compared with those in the IH group (*P*<0.05).

### Effects of Crude Lentinan on Short-Chain Fatty Acid Metabolism

**Figure 4:**
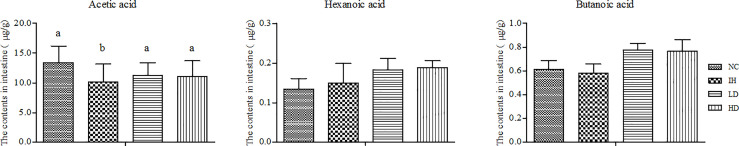
Changes of intestinal short-chain fatty acid (SCFA) contents after dietary 1% and 2% of CLNT in IHNV-infected rainbow trout (*P*<0.05). Different letters indicated significant differences among the groups.

SCFAs, including acetic acid, butanoic acid, and hexanoic acid, were detected from intestinal feces in the four experimental groups (**Figure 4**). IHNV infection notably decreased the acetic acid and butanoic acid contents compared with those in the NC group and slightly increased the hexanoic acid content. Supplementing with CLNT increased the levels of acetic acid, butanoic acid, and hexanoic acid compared with those in the IH group. Moreover, the supplement of CLNT slightly increased butanoic acid and hexanoic acid contents compared with those in the NC group. However, there was no significant difference in the changes of intestinal hexanoic acid and butanoic acid contents among the four groups (*P*>0.05). In summary, supplementary CLNT was conducive to stimulate the production of intestinal SCFAs in IHNV-infected rainbow trout.

### Effects of Crude Lentinan on Intestinal Microbiota

**Figure 5:**
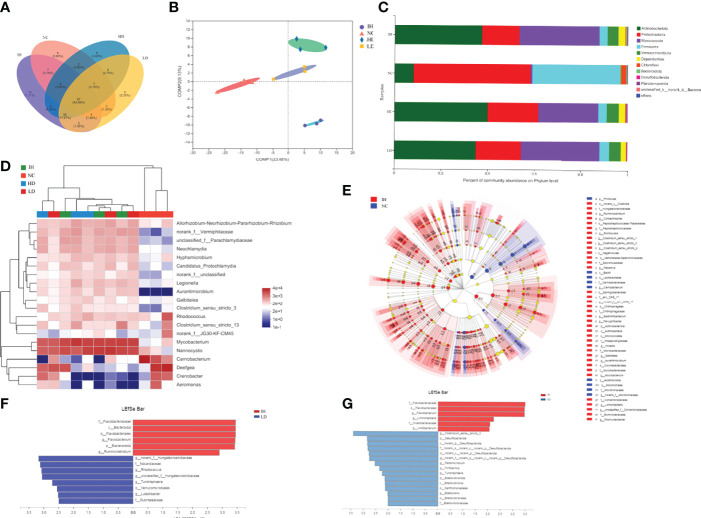
Effects of supplementary 1% and 2% of CLNT on intestinal microbiota in IHNV-infected rainbow trout. **(A)** OTU-Venn analysis of intestinal microbiota; **(B)** PLS-DA analysis of intestinal microbiota; **(C)** relative abundances of major phyla above 0.10% in at least one group; **(D)** community heat map analysis of top 20 genera in each group; **(E)** LDA Effect Size (LEfSe) analysis of microbes between NC and IH groups, LDA>4, *P*<0.05; **(F)** LEfSe analysis of microbes between LD and IH groups, LDA>2.5, *P*<0.05; **(G)** LEfSe analysis of microbes between HD and IH groups, LDA>2, *P*<0.05.

IHNV infection significantly elevated the intestinal microbiota community diversity compared to the NC group, represented by the relatively higher ACE, Chao1, and Shannon indices (*P*<0.05) (**Table 3**). Supplementary CLNT slightly increased intestinal microbiota community diversity. The evident group differences at the Operational Taxonomic Unit (OTU) level were observed among the four experimental groups (**Figure 5A**). The principal coordinates 1 and 2 respectively explained 23.08% and 9.13% of the microbial community structure variations (**Figure 5B**). In addition, the coverage indices were up to 99.80% of all species in the samples, which indicated a high quality of the assembled results. The data were deposited to the National Center for Biotechnology Information with accession No. PRJNA828277 (https://dataview.ncbi.nlm.nih.gov/object/PRJNA828277).

**Table 3:** Effects of supplementary 1% and 2% of CLNT on microbial alpha diversity in the intestine of IHNV-infected rainbow trout.

Groups	Indices				
	ACE	Chao1	Simpson	Shannon-wiener	Coverage
NC	194.35±26.94^a^	195.38±23.44^a^	0.47±0.34^a^	1.40±0.84^a^	99.92±0.02%
HD	300.08±33.20^b^	296.42±28.82^b^	0.19±0.05^b^	2.56±0.21^b^	99.88±0.04%
LD	306.10±32.87^b^	302.61±38.32^b^	0.22±0.05^b^	2.30±0.11^b^	99.86±0.02%
IH	300.32±46.21^b^	292.15±37.75^b^	0.23±0.04^b^	2.15±0.60^b^	99.91±0.03%

Different letters indicated significant differences between groups (P < 0.05).

At the phylum level, Proteobacteria was the most abundant in the NC group followed by Firmicutes and Actinobacteria (**Figure 5C**). IHNV infection significantly enriched intestinal Actinobacteria and Myxococcota in rainbow trout. Actinobacteria became the predominant phylum followed by Myxococcota and Proteobacteria in the IH group (*P*<0.05). Supplementary CLNT evidently reshaped the intestinal microbial compositions, represented by the increases in the proportions of Proteobacteria and Firmicutes compared with those in the IH group, as well as the decrease of Myxococcota. At the genus level, IHNV infection significantly increased the abundances of *Nannocystis* and *Mycobacterium* by 33.85% and 33.01% and decreased *Deefgea* by 27.13% compared with those in NC group, respectively (*P*<0.05) (**Figure 5D**). *Nannocystis* became the predominant genus followed by *Mycobacterium* and *Deefgea* in the IH group. *Unclassified-f-Parachlamydiaceae* and *norank-f-Vermiphilaceae* were newly emerged genera after IHNV infection. Supplementary CLNT decreased the relative abundances of *Mycobacterium* and *Nannocystis* compared with the IH group.

As shown in **Figure 5E**, the dominant microbes in the NC and IH groups were in a different phylum [linear discriminant analysis score (LDA)>2, *P*<0.05]. *Carnobacterium* was the most abundant phenotypic biomarker in healthy rainbow trout (NC group) and *Mycobacterium* in IHNV-infected ones (IH group). In addition, Flavobacterium was the specific taxon in the IH group and norank*-f-*Hungateiclostridiaceae in the LD group (**Figure 5F**), as well as Clostridium-sensu-stricto-3 in the HD group (**Figure 5G**) when the two groups were compared, respectively. The above results revealed that the differential enrichment of intestinal microbiota is closely related to the healthy status of rainbow trout.

### Effects of Crude Lentinan on Intestinal Microbial Metabolism

**Figure 6:**
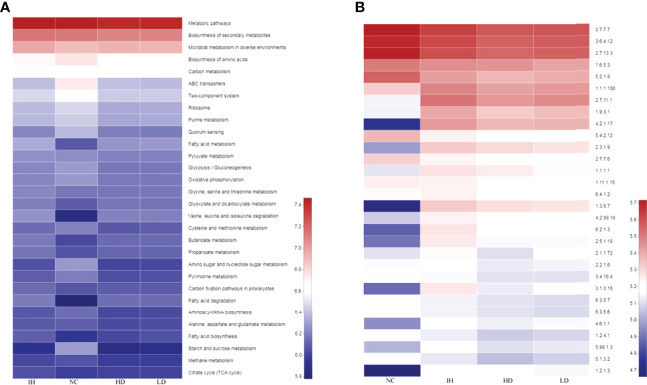
Predictions of metabolic pathways and enzymes within the intestinal microbiota. **(A)** Predicted metabolic pathways within the intestinal microbiota; **(B)** predicted enzymes within the microbiota.

Metabolism was the most important pathway within the intestinal microbiota (**SFigure1**). IHNV infection evidently increased bacterial distributions mapped to fatty acid metabolism, valine, leucine and isoleucine degradation, and fatty acid degradation, and declined the distributions in the biosynthesis of amino acids, glycolysis/gluconeogenesis, amino sugar and nucleotide sugar metabolism, etc. compared with NC group (**Figure 6A**). Supplementary CLNT decreased bacterial distributions in the biosynthesis of amino acids, carbon metabolism, purine metabolism, and so on, compared with those in the IH group.

In terms of enzymes, IHNV infection notably increased bacterial distributions in non-specific serine/threonine protein kinase (2.7.11.1), cytochrome-c oxidase (1.9.3.1), enoyl-CoA hydratase (4.2.1.17), acetyl-CoA C-acetyltransferase (2.3.1.9), DNA-(apurinic or apyrimidinic site) lyase (4.2.99.18), medium-chain acyl-CoA dehydrogenase (1.3.8.7), and long-chain fatty acid:CoA ligase (6.2.1.3) compared with those in the NC group (**Figure 6B**). There were trivial changes in the distributions of microbes that were mapped to the above enzymes in the LD and HD groups. Furthermore, IHNV infection significantly increased the bacterial distributions in DNA-methyltransferase (2.1.1.72), acetolactate synthase (2.2.1.6), protein-serine/threonine phosphatase (3.1.3.16), adenylate cyclase (4.6.1.1), and DNA topoisomerase (5.99.1.3) compared with those in the NC group (*P*<0.05). Supplementing with CLNT decreased bacterial distributions in these enzymes compared with those in the IH group.

### Redundancy Analysis

**Figure 7:**
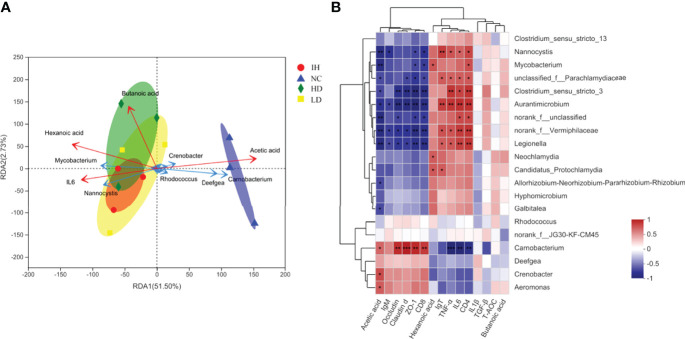
RDA and Spearman correlation analysis. **(A)** Redundancy analysis of key factors responding to top 20 gena; **(B)** Spearman correlation between key factors and the top 20 gena. The symbol "*,** and ***" indicated the significance between the key factor and genus at P≤0.05, P≤0.01 and P≤0.001, respectively.

Low collinearity of factors, including acetic acid, butanoic acid, hexanoic acid, and IL6, were screened from 15 indicators with the variable importance in projection (VIP) <4.0. The top 20 gena responding to the samples and these four indicators were shown in **Figure 6A**. The results of the RDA analysis indicated that the first two canonical axes explained 51.50% and 2.73% of the variation at the genus level, respectively. *Carnobacterium* and *Deefgea* exhibited relatively higher abundances in the healthy rainbow trout intestine, while *Mycobacterium* and *Nannocystis* in IHNV-infected ones (**Figure 7A**). These results were in accord with the analysis of intestinal microbiota compositions. Together with the Spearman correlation analysis, it was demonstrated that the distribution of *Carnobacterium* was positively correlated with the changes of acetic acid, claudin d, ZO-1, occludin, and CD8 (**Figure 7B**). The distributions of *unclassified-f-Parachalamydiaceae*, *Nannocystis*, *Aurantimicrobium*, *Clostridium_sensu_stricto_3*, *norank_f_Vermiphilaceae*, and *Legionella* were significantly positively correlated with the changes of IgT, TNF-α, IL6, and CD4 genes. The above results indicated that these parameters and their interactions impacted most on the distribution of 20 gena.

## Discussion

Viral diseases affect bony fish that can occur immediately and cause overwhelming deaths in the majority of farmed fish (23). The defense responses through the use of immunostimulants could be efficient to suppress the viral infection in fish to an extent (29, 30). Numerous studies reported that β-glucan could enhance the resistance to bacterial infection in fish like *Yersinia ruckeri*, *Flavobacterium columnare*, *Aeromonas salmonicida*, and *A. hydrophila* (29, 31, 32). Its resistance to viral diseases has also been reported such as grass carp hemorrhage virus, viral hemorrhagic septicemia virus, and nervous necrosis virus (33; 30, 34). Nevertheless, no information was available concerning the effects of lentinan on IHNV infection *in vivo*. Some studies on crude polysaccharides have proven their encouraging effects on immune enhancement (35–38). Considering the large population size of fish and the high cost of purified operation for polysaccharides, CLNT was selected to explore its antiviral effect on IHNV in this study. The results showed that CLNT well decreased the mortality of IHNV-infected rainbow trout, indicating that CLNT had the prophylactic effects on IHNV infection to an extent. The better efficiency at a low dosage of CLNT was possibly the result of multiple factors including the action of mode, dosage, and timing of administration, virus virulence, water temperature, fish species, and size.

Claudin d, occludin, and ZO-1 are tight junction proteins that are mainly responsible for controlling the initiation and stabilization of cel–cell adhesion and the paracellular passage of ions and solutes between cells (39). Their decreases could not only destroy intestinal integrity but also cause the cross of harmful non-self antigens and attack the host afterward (11). The low levels of claudin d, occludin, and ZO-1 in the IH group indicated that IHNV increased intestinal permeability. Intestinal imperfection could further weaken immunological tolerance accompanied by the release of a large number of inflammatory cytokines for responding to the damages. In this study, IHNV destroyed intestinal immune defense and consequently caused the increments in the expressions of TNF-α, IL-6, IL1β, and TGF-β genes in the intestine of rainbow trout. Similar results were found after the infection of infectious pancreatic necrosis virus or infectious salmon anemia virus (40, 41). Overproductions of these pro-inflammatory cytokines, especially TNF-α and IL-6, could further stimulate the other cytokines’ secretion and exacerbate the damage to tissues (42, 43). In this study, CLNT significantly increased the expressions of tight junction protein genes including claudin d, occludin, and ZO-1, as well as decreased TNF-α, IL-6, IL1β, and TGF-β genes (*P*<0.05), indicating that supplementary CLNT could improve the intestinal integrity and consequently ameliorate the intestinal inflammatory injury caused by IHNV infection. Moreover, T-AOC and SOD levels were significantly increased, and MDA levels were significantly decreased in the LD and HD groups (*P*<0.05), which suggested that CLNT has the capability to elevate the intestinal antioxidant state and then relieve the oxidative damage induced by IHNV infection to an extent. More importantly, IHNV-N and IHNV-L genes were significantly downregulated after CLNT administration (*P*<0.05), which suggested that CLNT effectively inhibited the viral replication in the intestine of IHNV-infected rainbow trout.

The adaptive immune response begins with the activation of CD4^+^ and CD8^+^ T lymphocytes (44). As we commonly know, the CD4^+^ T cells are increased after a viral infection. However, naive CD4^+^ T-cell activation could result in autoimmunity, which causes the hyperstimulation of the immune system and increases the disease burden in a host (45). In this study, CLNT downregulated the expression of the virus-specific CD4 gene, which was possibly related to its immunomodulatory effects on intestinal dysfunction caused by IHNV. There was no significant difference in the expression of the CD8 gene among the IH, LD, and HD groups (*P*>0.05). However, the changes of CD4 and CD8 genes might be attributed to the sampling time in this study. Immunoglobulins (Igs) are the vital index that specializes in reflecting the status of intestinal mucosal immunity. Some researchers reported that infectious pancreatic necrosis virus infection significantly increased IgT and IgM^+^ B-cell numbers in the foregut of rainbow trout in 7 days post-infection (*P*<0.05), viral hemorrhagic septicemia virus infection elevated the expressions of intestinal IgT and IgM genes in 6 days post-infection (46, 47). IHNV persistently induced the expression of IgT gene but inhibited IgM gene in 14 days post-infection in this study. It might be because IgT might exert the long-term immunity and immunological memory, and IgM provides an early-stage response during a viral infection (48). Interestingly, the supplement with CLNT significantly declined the expression of IgT gene (*P*<0.05) but slightly increased IgM. It was speculated that these interventions could be conducive to assuring the homeostasis between sIgs and microbiota (22). Indeed, the secretion of IgT directly affects the intestinal microbiota structure (49). Meanwhile, the upregulation of IgM was possibly due to the immunomodulation of CLNT to the B-cell antigen receptor (BCR) in IgM^+^ B cells (50). In summary, CLNT significantly inhibited intestinal IHNV infection through strengthening the intestinal immune barrier in rainbow trout (*P*<0.05). However, the responses of immune factors triggered by CLNT could be closely related to the pathogenesis of IHNV and reflected the mechanism of viruses for dealing with the host responses.

The intestinal tract of fish is generally recognized as a major immune organ, where it is inhabited by a vast population of microbes, playing important roles in pathogen protection, nutritional, endocrine, immune, and physiological functions (51). Proteobacteria are recognized as the predominant phylum in most marine and freshwater fish. On the one hand, some members could adhere to epithelial linings and induce inflammatory signaling; on the other hand, members participate in synthesizing fatty acids (52). Meanwhile, Firmicutes, like *Carnobacterium* and *Clostridium* as the main lactic acid producer, plays the preventive effects against the development of intestinal diseases (53, 54). In this study, the dominant phylum was Proteobacteria in healthy rainbow trout intestine followed by Firmicutes, which was in accordance with the previous studies (55, 56). This balance of intestinal microbiota ensured the normal operation of intestinal immune response in rainbow trout. Similar to the spring viremia of carp virus (57), IHNV significantly increased the intestinal microbial α diversity in rainbow trout (*P*<0.05). *Mycobacteria* (Actinobacteria) and *Nannocystis* (Myxococcota) are prevalent in soil and aquatic environments and are well known for causing disease in animals and humans (58, 59). They might cause local hypoxia and further result in the tissue oxidative injury (60). The aberrant decreases in the abundance of *Carnobacterium* and *Deefgea* as well as increases of *Nannocystis* and *Mycobacterium* proved that IHNV significantly disrupted the intestinal microbiota homeostasis. Supplementing with CLNT effectively promoted the growth of beneficial microbes and inhibited the detrimental ones, indicating that CLNT exerted the prebiotic roles in the intestinal microbiota of IHNV-infected rainbow trout.

In the complex interaction between host and viruses, viruses can manipulate and alter the metabolic state of the host for their efficient survival. Several studies revealed that the cellular fatty acid metabolism is necessary for the viral replication such as Kaposi’s sarcoma–associated, herpesvirus hepatitis C virus, and human cytomegalovirus (61–63). Generally, the occurrence of metabolism depends on the dominance of microbial proportion. Medium-chain acyl-CoA dehydrogenase and long-chain fatty acid:CoA ligase are mainly implicated in the production of long-chain fatty acids (LCFAs) that are the main component of the viral lipid membrane of enveloped viruses (62). IHNV, as the enveloped virus, promoted the overgrowth of microbes mapped to the two enzymes, which suggested that IHNV might cunningly hijack these microbes to produce the LCFAs to satisfy the replication for themselves. However, supplementing with CLNT did not inhibit the growth of microbes mapped to the above enzymes, which was probably due to these microbes being involved in the fatty acid metabolic process after the digestion of CLNTs. Enoyl-CoA hydratase and acetyl-CoA C-acetyltransferase are the important players for fatty acid degradation, which could catalyze the acetic acid to synthesize fatty acid and cholesterol (52). Fatty acid and cholesterol are the essential components for establishing a functional virus replication factory (64). In the present study, the virus induced the significant changes of bacterial distributions mapped to the enzymes, which suggested that IHNV took use of intestinal microbes to drive the specific metabolic pathways to acquire the substrates and energy for its survival (*P*<0.05). It is well known that polysaccharides are not directly digested but are processed to biologically active metabolites, particularly SCFAs by intestinal microbes (25). Indeed, supplementing with CLNT increased the intestinal acetic acid, butanoic acid, and hexanoic acid. It means that CLNT exerted the substrate benefits for intestinal SCFA–producing bacteria and further boosted their participation in SCFA metabolisms. The production of SCFAs are rapidly absorbed by the intestinal epithelium, or function with their receptors on the surface of intestinal mucosal T cells to ensure the intestinal immune barrier and reduce the intestinal pH to inhibit the destructive bacterial growth (24, 65). Furthermore, CLNT significantly decreased the propagation of microbes mapped to the enzymes that are involved in the biosynthesis of valine, isoleucine, and leucine (BCAAs) (*P*<0.05). It was reported that BCAAs were critical for viral particle formation (hepatitis C virus) (66). Likewise, the overgrowth of microbes that implicated the biosynthesis BCAAs induced by IHNV was probably related to their replication. Collectively, CLNT reshaped the intestinal microbiota and prevented IHNV infection, which might benefit from the enhancement of immune surveillance induced by the metabolites produced by intestinal microbiota, such as SCFAs.

In conclusion, supplementation of dietary CLNT significantly declined the mortality of rainbow trout caused by IHNV infection (*P*<0.05). CLNT effectively inhibited the IHNV invasion in rainbow trout through improving the intestinal integrity, suppressing the intestinal inflammatory responses, and ameliorating the intestinal oxidative damage. Supplementary CLNT reshaped intestinal microbial compositions in IHNV-infected rainbow trout. At the genus level, CLNT promoted the growths of *Carnobacterium* and *Deefgea* and inhibited the *Mycobacterium* and *Nannocystis*. Moreover, supplementing with CLNT rectified the self-serving metabolic pathways of intestinal microbiota induced by IHNV, such as fatty acid metabolism, and consequently promoted the production of SFCAs in the intestine. The results showed that CLNT effectively protected the rainbow trout from IHNV infection by improving the intestinal immune barrier and microbiota and promoting the production of intestinal SFCAs. These findings indicated that CLNT could be potentially used as an immunostimulant in fish feedstuff for preventing IHNV infection in rainbow trout.

## Data Availability Statement

The datasets presented in this study can be found in online repositories. The name of the repository and accession number can be found below: National Center for Biotechnology Information (NCBI) BioProject, https://www.ncbi.nlm.nih.gov/bioproject/, PRJNA828277.

## Ethics Statement

The animal study was reviewed and approved by Animal Welfare Committee of Heilongjiang River Fishery Research Institute Chinese Academy of Fishery Sciences.

## Author Contributions

GR: writing-original draft, writing-review and editing, designed research studies, experimentation and data acquisition, performing experiments, and data analysis. LX: designed research studies, conceptualization, and methodology. JZ: software and formal analysis. YS: software and formal analysis. XC: software and formal analysis. TL: resources and funding acquisition. QZ: resources, writing-reviewing, and editing. All authors contributed to the article and approved the submitted version.

## Funding

This work was supported by the National Key Research and Development Program of China (2019YFE0115500), National Natural Science Foundation of China (32002437), and Central public-interest Scientific Research Institution Basal Research Fund, Chinese Academy of Fishery Sciences (HSY202106M).

## Conflict of Interest

The authors declare that the research was conducted in the absence of any commercial or financial relationships that could be construed as a potential conflict of interest.

## Publisher’s Note

All claims expressed in this article are solely those of the authors and do not necessarily represent those of their affiliated organizations, or those of the publisher, the editors and the reviewers. Any product that may be evaluated in this article, or claim that may be made by its manufacturer, is not guaranteed or endorsed by the publisher.

## References

[1] Garver KA Troyer RM Kurath G. Two Distinct Phylogenetic Clades of Infectious Hematopoietic Necrosis Virus Overlap Within the Columbia River Basin. Dis Aquat Org (2003) 55:187–203. doi: 10.3354/dao055187 13677505

[2] Kurath G Garver KA Troyer RM Emmenegger EJ Einer-Jensen K Anderson ED. Phylogeography of Infectious Haematopoietic Necrosis Virus in North America. J Gen Virol (2003) 84:803–14. doi: 10.1099/vir.0.18771-0 12655081

[3] Kim CH Dummer DM Chiou PP Leong JC. Truncated Particles Produced in Fish Surviving Infectious Hematopoietic Necrosis Virus Infection: Mediators of Persistence? J Virol (1999) 73:843–9. doi: 10.1128/JVI.73.1.843-849.1999 PMC103901 9847400

[4] Miller K Traxler G Kaukinen K Li S Richard J Ginther N. Salmonid Host Response to Infectious Hematopoietic Necrosis (IHN) Virus: Cellular Receptors, Viral Control, and Novel Pathways of Defence. Aquaculture (2007) 272:S217–37. doi: 10.1016/j.aquaculture.2007.08.041

[5] Bootland LM Leong J. Infectious Haematopoietic Necrosis Virus. In: Woo PTK Bruno DW, editors. Fish Diseases and Disorders. Viral, Bacterial and Fungal Infections. UK: CABI Publishing (1999). p. 57–121.

[6] Vetvicka V Vannucci L Sima P. The Effects of β-Glucan on Fish Immunity. North Am J Med Sci (2013) 5(10):580–8. doi: 10.4103/1947-2714.120792 PMC3842698 24350069

[7] Khan I Huang GX Li XA Leong W Xia WR Hsiao WLW. Mushroom Polysaccharides From Ganoderma Lucidum and *Poria Cocos* Reveal Prebiotic Functions. J Funct Foods (2018) 41:191–201. doi: 10.1016/j.jff.2017.12.046

[8] Suga Y Takehana K. Lentinan Diminishes Apoptotic Bodies in the Ileal Crypts Associated With S-1 Administration. Int Immunopharmacol (2017) 50:55–60. doi: 10.1016/j.intimp.2017.06.012 28628771

[9] Andrew M Jayaraman G. Marine Sulfated Polysaccharides as Potential Antiviral Drug Candidates to Treat Corona Virus Disease (COVID-19). Carbohydr Res (2021) 505:108326. doi: 10.1016/j.carres.2021.108326 34015720 PMC8091805

[10] Djordjevic B Škugor S Jørgensen SM Øverland M Mydland LT Krasnov A. Modulation of Splenic Immune Responses to Bacterial Lipopolysaccharide in Rainbow Trout (*Oncorhynchus Mykiss*) Fed Lentinan, a Beta-Glucan From Mushroom Lentinula Edodes. Fish Shellfish Immunol (2009) 26:201–9. doi: 10.1016/j.fsi.2008.10.012 19010422

[11] Nishitani Y Zhang L Yoshida M Azuma T Kanazawa K Hashimoto T. Intestinal Anti-Inflammatory Activity of Lentinan: Influence on IL-8 and TNFR1 Expression in Intestinal Epithelial Cells. PloS One (2013) 8(4):e62441. doi: 10.1371/journal.pone.0062441 23630633 PMC3632531

[12] Zhang Y Liu Y Zhou Y Zheng Z Tang W Song M. Lentinan Inhibited Colon Cancer Growth by Inducing Endoplasmic Reticulum Stress-Mediated Autophagic Cell Death and Apoptosis. Carbohydr Polymers (2021) 267:118154. doi: 10.1016/j.carbpol.2021.118154 34119128

[13] Xu X Zhang X. *Lentinula Edodes*-Derived Polysaccharide Alters the Spatial Structure of Gut Microbiota in Mice. PLoS One (2015) 10(1):e0115037. doi: 10.1371/journal.pone.0115037 25608087 PMC4301806

[14] Drolet BS Rohovec JS Leong JC. The Route of Entry and Progression of Infectious Haematopoietic Necrosis Virus in *Oncorhynchus Mykiss* (Walbaum): A Sequential Immunohistochemical Study. J Fish Dis (1994) 17:337–44. doi: 10.1111/j.1365-2761.1994.tb00229.x

[15] Dixon P Paley R Alegria-Moran R Oidtmann B. Epidemiological Characteristics of Infectious Hematopoietic Necrosis Virus (IHNV): A Review. Vet Res (2016) 47:63. doi: 10.1186/s13567-016-0341-1 27287024 PMC4902920

[16] OIE. World Organization for Animal Health, Aquatic Animal Health Code (2016). Available at: http://www.oie.int/international-standardsetting/aquatic-code/access-online.

[17] Ahmadivand S Soltani M Mardani K Shokrpoor S Hassanzadeh R Ahmadpoor M. Infectious Hematopoietic Necrosis Virus (IHNV) Outbreak in Farmed Rainbow Trout in Iran: Viral Isolation, Pathological Findings, Molecular Confirmation, and Genetic Analysis. Virus Res (2017) 229:17–23. doi: 10.1016/j.virusres.2016.12.013 28012997

[18] Hansen JD La Patra SE. Induction of the Rainbow Trout MHC Class I Pathway During Acute IHNV Infection. Immunogenetics (2002) 54:654–61. doi: 10.1007/s00251-002-0509-x 12466898

[19] Landis ED Purcell MK Thorgaard GH Wheeler PA Hansen JD. Transcriptional Profiling of MHC Class I Genes in Rainbow Trout Infected With Infectious Hematopoietic Necrosis Virus. Mol Immunol (2008) 45(6):1646–57. doi: 10.1016/j.molimm.2007.10.003 18187194

[20] Bledsoe JW Ma J Cain K Bruce TJ Rawles A Abernathy J. Multi-Tissue RNAseq Reveals Genetic and Temporal Differences in Acute Response to Viral (IHNV) Infection Among Three Selected Lines of Rainbow Trout With Varying Resistance. Fish Shellfish Immunol (2022) 124:343–61. doi: 10.1016/j.fsi.2022.03.034 35398222

[21] Alghetaa H Mohammed A Zhou J Singh N Nagarkatti M Nagarkatti P. Resveratrol-Mediated Attenuation of Superantigen-Driven Acute Respiratory Distress Syndrome Is Mediated by Microbiota in the Lungs and Gut. Pharmacol Res (2021) 167:105548. doi: 10.1016/j.phrs.2021.105548 33722710 PMC10116750

[22] Salinas I Fernández-Montero Á Ding Y Sunyer JO. Mucosal Immunoglobulins of Teleost Fish: A Decade of Advances. Dev Comp Immunol (2021) 121:104079. doi: 10.1016/j.dci.2021.104079 33785432 PMC8177558

[23] Dong S Ding L Cao J Liu X Xu H Meng K. Viral-Infected Change of the Digestive Tract Microbiota Associated With Mucosal Immunity in Teleost Fish. Front Immunol (2019) 10:2878. doi: 10.3389/fimmu.2019.02878 31921142 PMC6930168

[24] Wu T Shen M Yu Q Chen Y Chen X Yang J. *Cyclocarya Paliurus* Polysaccharide Improves Metabolic Function of Gut Microbiota by Regulating Short-Chain Fatty Acids and Gut Microbiota Composition. Food Res Int (2021) 141:110119. doi: 10.1016/j.foodres.2021.110119 33641986

[25] Fang Q Hu Y Nie JL Nie SP. Effects of Polysaccharides on Glycometabolism Based on Gut Microbiota Alteration. Trends Food Sci Technol (2019) 92:65–70. X Q. doi: 10.1016/j.tifs.2019.08.015

[26] Alwin A Karst SM. The Influence of Microbiota-Derived Metabolites on Viral Infections. Curr Opin Virol (2021) 49:151–6. doi: 10.1016/j.coviro.2021.05.006 PMC9447419 34144380

[27] Ren G Xu L Lu T Yin J. Structural Characterization and Antiviral Activity of Lentinan From *Lentinus Edodes* Mycelia Against Infectious Hematopoietic Necrosis Virus. Int J Biol Macromolecules (2018) 115:1202–10. doi: 10.1016/j.ijbiomac.2018.04.132 29704603

[28] Zhao J Xu L Ren G Dong Y Cao Y Shao Y. Complete Genome Sequence and Phylogenetic Analysis of Sn1203 Strain of Infectious Hematopoietic Necrosis Virus. Chin J Fisheries (2020) 33(2):1–9. doi: 1005-3822(2020)02-0001-09

[29] Lopes LMF de Mello MMM Urbinati EC. β-Glucan Reduces Cortisol Plasma Levels, Enhances Innate Immune System After A. *Hydrophila* Inoculation, and has Lipolytic Effects on the Pacu *(Piaractus Mesopotamicus*). Aquaculture (2022) 546:737411. doi: 10.1016/j.aquaculture.2021.737411

[30] Krishnan R Jang Y Oh M. Beta Glucan Induced Immune Priming Protects Against Nervous Necrosis Virus Infection in Sevenband Grouper. Fish Shellfish Immunol (2022) 121:163–71. doi: 10.1016/j.fsi.2022.01.005 35017048

[31] Amphan S Unajak S Printrakoon C Areechon N. Feeding-Regimen of β-Glucan to Enhance Innate Immunity and Disease Resistance of Nile Tilapia, *Oreochromis Niloticus* Linn., Against *Aeromonas Hydrophila* and *Flavobacterium Columnare* . Fish Shellfish Immunol (2019) 87:120–8. doi: 10.1016/j.fsi.2018.12.062 30597253

[32] Akhtar MS Tripathi PH Pandey A Ciji A. β-Glucan Modulates Non-Specific Immune Gene Expression, Thermal Tolerance and Elicits Disease Resistance in Endangered Tor Putitora Fry Challenged With Aeromonas Salmonicida. Fish Shellfish Immunol (2021) 119:154–62. doi: 10.1016/j.fsi.2021.09.038 34597814

[33] Beaulaurier J Hershberger PK. Susceptibility of Pacific Herring to Viral Hemorrhagic Septicemia Is Influenced by Diet. J Aquat Anim Health (2012) 24(1):43–8. doi: 10.1080/08997659.2012.668511 22779213

[34] Kim Y Ke F Zhang Q. Effect of β-Glucan on Activity of Antioxidant Enzymes and Mx Gene Expression in Virus Infected Grass Carp. Fish Shellfish Immunol (2009) 27(2):336–40. doi: 10.1016/j.fsi.2009.06.006 19540347

[35] Tan J McKenzie C Potamitis M Thorburn AN Mackay CR Macia L. The Role of Short-Chain Fatty Acids in Health and Disease. Adv Immunol (2014) 121:91–119. doi: 10.1016/B978-0-12-800100-4.00003-9 24388214

[36] Chaiyama V Keasompong S LeBlanc JG de LeBlanc AM Chatel J Chanput W. Action Modes of the Immune Modulating Activities of Crude Mushroom Polysaccharide From Phallus Atrovolvatus. Bioactive Carbohydr Dietary Fibre (2020) 23:100216. doi: 10.1016/j.bcdf.2020.100216

[37] Zhang W Gong L Liu Y Zhou Z Wan C Xu J. Immunoenhancement Effect of Crude Polysaccharides of *Helvella Leucopus* on Cyclophosphamide-Induced Immunosuppressive Mice. J Funct Foods (2020) 69:103942. doi: 10.1016/j.jff.2020.103942

[38] Muttharasi C Gayathri V Muralisankar T Mohan K Uthayakumar V Radhakrishnan S. Growth Performance, Digestive Enzymes and Antioxidants Activities in the Shrimp Litopenaeus Vannamei Fed With *Amphiroa Fragilissima* Crude Polysaccharides Encapsulated Artemia Nauplii. Aquaculture (2021) 545(15):737263. doi: 10.1016/j.aquaculture.2021.737263

[39] Hartsock A Nelson WJ. Adherens and Tight Junctions: Structure, Function and Connections to the Actin Cytoskeleton. Biochim Biophys Acta (BBA)-Biomembranes (2008) 1778(3):660–9. doi: 10.1016/j.bbamem.2007.07.012 PMC2682436 17854762

[40] García-Rosado E Markussen T Kileng O Baekkevold E Robertsen B Mjaaland S. Molecular and Functional Characterization of Two Infectious Salmon Anaemia Virus (ISAV) Proteins With Type I Interferon Antagonizing Activity. Virus Res (2008) 133:228–38. doi: 10.1016/j.virusres.2008.01.008 18304672

[41] Langevin C Aleksejeva E Passoni G Palha N Levraud J Boudinot P. The Antiviral Innate Immune Response in Fish: Evolution and Conservation of the IFN System. J Mol Biol (2013) 425(24):4904–20. doi: 10.1016/j.jmb.2013.09.033 24075867

[42] Tatiya-aphiradee N Chatuphonprasert W Jarukamjorn K. Ethanolic *Garcinia Mangostana* Extract and α-Mangostin Improve Dextran Sulfate Sodium-Induced Ulcerative Colitis *via* the Suppression of Inflammatory and Oxidative Responses in ICR Mice. J Ethnopharmacol (2021) 265:113384. doi: 10.1016/j.jep.2020.113384 32927006

[43] Santos JGO Migueis DP do Amaral JB Bachi ALL Boggi AC Thamboo A. Impact of SARS-CoV-2 on Saliva: TNF-α, IL-6, IL-10, Lactoferrin, Lysozyme, IgG, IgA, and IgM. J Oral Biosci (2022) 64(1):108–13. doi: 10.1016/j.job.2022.01.007 PMC8788095 35091065

[44] Imam T Park S Kaplan MH Olson MR. Effector T Helper Cell Subsets in Inflammatory Bowel Diseases. Front Immunol (2018) 9:1212. doi: 10.3389/fimmu.2018.01212 29910812 PMC5992276

[45] Lange PT White MC Damania B. Activation and Evasion of Innate Immunity by Gammaherpesviruses. J Mol Biol (2021) 434(6):167214. doi: 10.1016/j.jmb.2021.167214 34437888 PMC8863980

[46] Ballesteros NA Rodríguez Saint-Jean S Perez-Prieto SI Aquilino C Tafalla C. Modulation of Genes Related to the Recruitment of Immune Cells in the Digestive Tract of Trout Experimentally Infected With Infectious Pancreatic Necrosis Virus (IPNV) or Orally Vaccinated. Dev Comp Immunol (2014) 44:195–205. doi: 10.1016/j.dci.2013.12.009 24370535

[47] Leal E Ordás MC Soleto I Zarza C McGurk C Tafalla C. Functional Nutrition Modulates the Early Immune Response Against Viral Haemorrhagic Septicaemia Virus (VHSV) in Rainbow Trout. Fish Shellfish Immunol (2019) 94:769–79. doi: 10.1016/j.fsi.2019.09.070 31580935

[48] Gong S Ruprecht RM. Immunoglobulin M: An Ancient Antiviral Weapon-Rediscovered. Front Immunol (2020) 11:1943. doi: 10.3389/fimmu.2020.01943 32849652 PMC7432194

[49] Nakajima A Vogelzang A Maruya M Miyajima M Murata M Son A. IgA Regulates the Composition and Metabolic Function of Gut Microbiota by Promoting Symbiosis Between Bacteria. J Exp Med (2018) 215:2019–34. doi: 10.1084/jem.20180427 PMC6080902 30042191

[50] Chen J Lei Y Dong Z Fu S Li L Gao A. β-Glucan Could Also Participate in Immunomodulation Through BCR Receptor in IgM^+^ B Cells. Toxicol Vitro (2022) 81:105334. doi: 10.1016/j.tiv.2022.105334 35182770

[51] Vargas-Albores F Martínez-Córdova LR Hernández-Mendoza A Cicala F Lago-Lestón A Martínez-Porchas M. Therapeutic Modulation of Fish Gut Microbiota, a Feasible Strategy for Aquaculture? Aquaculture (2021) 544:737050. doi: 10.1016/j.aquaculture.2021.737050

[52] Rios-Covian D Ruas-Madiedo P Margolles A Gueimonde M de los Reyes-Gavilan CG Salazar N. Intestinal Short Chain Fatty Acids and Their Link With Diet and Human Health. Front Microbiol (2016) 7:185. doi: 10.3389/fmicb.2016.00185 26925050 PMC4756104

[53] Graf D Cagno RD Fåk R Flint HF Nyman M Saarela M. Contribution of Diet to the Composition of the Human Gut Microbiota. Microbial Ecol Health Dis (2015) 26:10. doi: 10.3402/mehd.v26.26164 PMC4318938 25656825

[54] Ganesan K Chung SK Vanamala J Xu B. Causal Relationship Between Diet-Induced Gut Microbiota Changes and Diabetes: A Novel Strategy to Transplant *Faecalibacterium Prausnitzii* in Preventing Diabetes. Int J Mol Sci (2018) 19(12):3720. doi: 10.3390/ijms19123720 PMC6320976 30467295

[55] Green TJ Smullen R Barnes AC. Dietary Soybean Protein Concentrate-Induced Intestinal Disorder in Marine Farmed Atlantic Salmon, Salmo Salar Is Associated With Alterations in Gut Microbiota. Vet. Microbiol (2013) 166(1-2):286–92. doi: 10.1016/j.vetmic.2013.05.009 23810699

[56] Ricaud K Rey M Plagnes-Juan E Larroquet L Even M Quillet E. Composition of Intestinal Microbiota in Two Lines of Rainbow Trout (*Oncorhynchus Mykiss*) Divergently Selected for Muscle Fat Content. Open Microbiol J (2018) 12(1):308–20. doi: 10.2174/1874285801812010308 PMC6142665 30288186

[57] Meng K Ding L Wu S Wu Z Cheng G Zhai X. Interactions Between Commensal Microbiota and Mucosal Immunity in Teleost Fish During Viral Infection With SVCV. Front Immunol (2021) 7(12):654758. doi: 10.3389/fimmu.2021.654758 PMC8058427 33897703

[58] Hruska K Kaevska M. Mycobacteria in Water, Soil, Plants and Air: A Review. Vet Sci (2012) 57:623–79. doi: 10.1093/ilar.53.3-4.370

[59] Murphy CL Yang R Decker T Cavalliere C Andreev V Bircher N. Genomes of Novel Myxococcota Reveal Severely Curtailed Machineries for Predation and Cellular Differentiation. Appl Environ Microbiol (2021) 87(23):e0170621. doi: 10.1128/AEM.01706-21 34524899 PMC8580003

[60] Harold LK Jinich A Hards K Cordeiro A Keighley LM Cross A. Deciphering Functional Redundancy and Energetics of Malate Oxidation in Mycobacteria. J Biol Chem (2022) 23:101859. doi: 10.1016/j.jbc.2022.101859 PMC9062433 35337802

[61] Delgado T Sanchez EL Camarda R Lagunoff M. Global Metabolic Profiling of Infection by an Oncogenic Virus: KSHV Induces and Requires Lipogenesis for Survival of Latent Infection. PLoS Pathogen (2012) 8(8):e1002866. doi: 10.1371/journal.ppat.1002866 22916018 PMC3420960

[62] Koyuncu E Purdy JG Rabinowitz JD Shenk T Damania B. Saturated Very Long Chain Fatty Acids Are Required for the Production of Infectious Human Cytomegalovirus Progeny. PLoS Pathog (2013) 9:e1003333. doi: 10.1371/journal.ppat.1003333 23696731 PMC3656100

[63] Meyers NL Fontaine KA Kumar GR Ott M. Entangled in a Membranous Web: ER and Lipid Droplet Reorganization During Hepatitis C Virus Infection. Curr Opin Cell Biol (2016) 41:117–24. doi: 10.1016/j.ceb.2016.05.003 PMC5477849 27240021

[64] Konan VK Sanchez-Felipe L. Lipids and RNA Virus Replication. Curr Opin Virol (2014) 9:45–52. doi: 10.1016/j.coviro.2014.09.005 25262061 PMC4625897

[65] Nearing JT Connors J Whitehouse S Van Limbergen J Macdonald T Kulkarni K. Infectious Complications Are Associated With Alterations in the Gut Microbiome in Pediatric Patients With Acute Lymphoblastic Leukemia. Front Cell Infect Microbiol (2019) 9:28. doi: 10.3389/fcimb.2019.00028 30838178 PMC6389711

[66] Ishida H Kato T Takehana K Tatsumi T Hosui A Nawa T. Valine, the Branched-Chain Amino Acid, Suppresses Hepatitis C Virus RNA Replication But Promotes Infectious Particle Formation. Biochem Biophys Res Commun (2013) 437(1):127–33. doi: 10.1016/j.bbrc.2013.06.051 23806690

