# Supplementary figures and images for: Supplementation of Dietary Crude Lentinan Improves the Intestinal Microbiota and Immune Barrier in Rainbow Trout (Oncorhynchus mykiss) Infected by Infectious Hematopoietic Necrosis Virus
Source: Front Immunol. 2022 Jun 22;13:920065. doi: 10.3389/fimmu.2022.920065 (PMC9258421; doi:10.3389/fimmu.2022.920065)

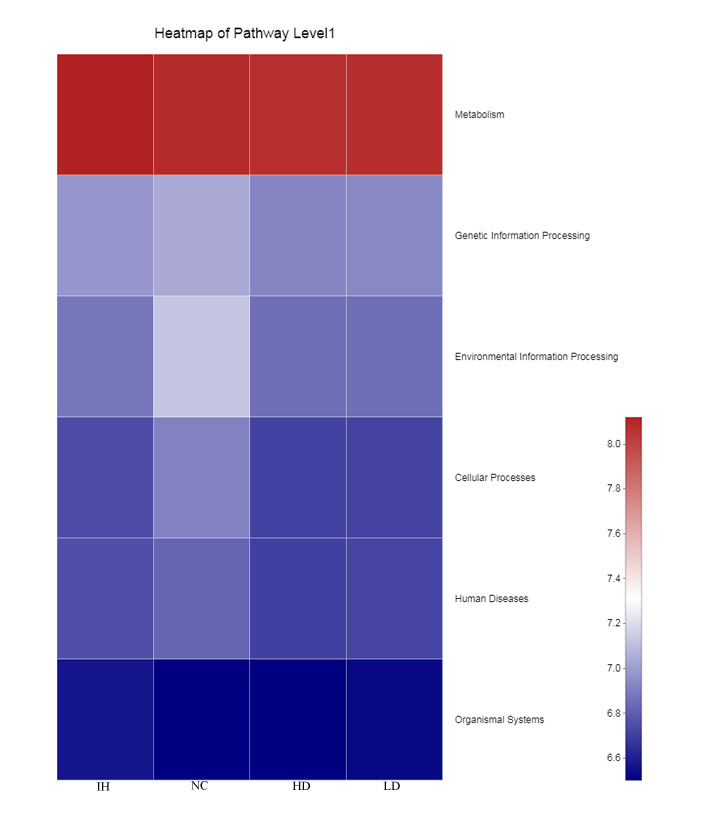

Supplement: Supplementary file 1 [file Image_1.jpeg]
