# Supplementary material for: Supplementation of Dietary Crude Lentinan Improves the Intestinal Microbiota and Immune Barrier in Rainbow Trout (Oncorhynchus mykiss) Infected by Infectious Hematopoietic Necrosis Virus
Source: Front Immunol. 2022 Jun 22;13:920065. doi: 10.3389/fimmu.2022.920065 (PMC9258421; doi:10.3389/fimmu.2022.920065)
Supplement: Supplementary file 2 [file Table_1.doc]

STable 1 Primer sequences for RT-qPCR analysis

| Primers | Primer, forward/reverse (5’ to 3’) | Accession Number |
| --- | --- | --- |
| ZO-1 | F: ACCACCACCTCTTCACCACCACCA  R: TGCCCTCTGACACCCAGACCAACT | XM_036969356.1 |
| Occludin | F: GCTTTGGTGGTGCTGCCTATGGTG  R: AATCCTTTGCCAGTGCGAGGGTCA | NM_001190446.1 |
| Claudin d | F: ACCCAAGTACAAAGAACC  R: AGCCCACTGAGGAACAC | NM 001165403.1 |
| TGF-β | F: GGGAAACCAATGGAGGA  R: GCTAAGCTGGCTGTGGC | X99303.1 |
| TNF-α | F: TCTTACCGCTGACACAGTGC  R: AGAAGCCTGGCTGTAAACGA | AJ277604.1 |
| IL1β | F: GGAGAGGTTAAAGGGTGGCGA  R: TGCCGACTCCAACTCCAACA | NM01123582.1 |
| IL6 | F: CCTTGCGGAACCAACAGTTTG  R: CCTCAGCAACCTTCATCTGGTC | HG974247.1 |
| CD4 | F: CCTGCTCATCCACAGCCTAT  R: CTTCTCCTGGCTGTCTGACC | AY973030.1 |
| CD8 | F: AGTCGTGCAAAGTGGGAAAG  R: /GGTTGCAATGGCATACAGTC | NM_001124263 |
| IgM | F: ACCTTAACCAGCCGAAAGGG  R: TGTCCCATTGCTCCAGTCC | X65263.1 |
| IgT | F: AGCACCAGGGTGAAACCA  R: GCGGTGGGTTCAGAGTCA | AY870265 |
| IHNV-N | F: GCTCACCAAGGCTGTTTAT  R: CATCAGTCTTACAATGCGTCTA | MT242597.1 |
| IHNV-L | F:TGGGAGCCATTGGTGATT  R: GGTTGAGCGTCGGTTTGC | MT242597.1 |
| β-actin | F: CTGTTGGCTTTGGGGTTGAG  R: CAGGGAGTGATGGTTGGGATG | AF254414.1 |
